# Left ventricular short-axis systolic function changes in patients with hypertrophic cardiomyopathy detected by two-dimensional speckle tracking imaging

**DOI:** 10.1186/s12872-018-0753-0

**Published:** 2018-01-30

**Authors:** Jun Huang, Zi-Ning Yan, Yi-Fei Rui, Li Fan, Chang Liu, Jie Li

**Affiliations:** Department of Echocardiography, the Affiliated Changzhou No.2 People’s Hospital of Nanjing Medical University, Changzhou, China

**Keywords:** Two-dimensional speckle tracking imaging, Hypertrophic cardiomyopathy, Circumferential, Radial, Strain

## Abstract

**Background:**

Hypertrophic cardiomyopathy (HCM) is a genetic disease was characterised by left ventricular hypertrophy (LVH), myocardial fibrosis, fiber disarray. The short-axis systolic function is important in left ventricle function.

**Methods:**

Forty one healthy subjects and 37 HCM patients were enrolled for this research. Parasternal short-axis at the basal, middle, and apical levels were acquired by Echocardiography. The peak systolic circumferential strain of the endocardial, the middle and the epicardial layers, the peak systolic radial strain, and the peak systolic rotational degrees at different short-axis levels were measured by 2-dimensional speckle tracking imaging (2D–STI).

**Results:**

The peak systolic circumferential strain of the septum and anterior walls in HCM patients was significantly lower than normal subjects. All of the peak systolic radial strain in HCM patients was significantly lower than normal subjects. The rotational degrees at the base and middle short-axis levels in HCM patients were larger than normal subjects. The interventricular septal thickness in end-diastolic period correlated to the peak systolic circumferential strain of the septum wall.

**Conclusions:**

The short-axis systolic function was impaired in HCM patients. The peak circumferential systolic strain of the different layers, peak systolic radial strain and rotation degrees of the different short-axis levels detected by 2D–STI are very feasible for assessing the short-axis function in HCM patients.

## Background

Hypertrophic cardiomyopathy (HCM) is a common cardiac disease [1, 2]. As the universal use of the echocardiography, computer tomography, and magnetic resonance imaging, the discovery of HCM have increased annually [3–6]. The disease is characterized by thickening the ventricular myocardium walls [7]. It is a genetic disorder of the myocardium caused by mutations in cardiac sarcomeric proteins. The pathology of HCM is the gross of the cardiac myocardial hypertrophy and fiber disarray. HCM patients often asymptomatic throughout life, but someone may have severe symptoms like sudden cardiac death at a young age [8, 9].

Two-dimensional speckle tracking imaging (2D–STI) can assess myocardial function accurately [10]. Currently, researches mainly focussed on myocardial function by detected the global myocardial strain, strain rate and torsion [11–16]. As we know, a normal myocardium is contained three layers: endocardial, middle myocardial and epicardial layers [17, 18]. Endocardial and epicardial layers are longitudinal oriented, and the middle myocardial is circumferential oriented. When the longitudinal and circumferential myocardium contract and relax, the cardiac myocardium deformation occurs in three directions: longitudinally, circumferentially, and radially. Our previous study showed that in HCM patients, the longitudinal function was damaged, even with normal LV ejection fraction [19], however, short-axis cardiac function as circumferentially and radially is also essential like longitudinally function. So, in this study, we mainly analysed the short-axis function in HCM patients.

Of data, detect the peak circumferential systolic strain of endocardial, middle myocardial, and epicardial layers in HCM patients is rare. The innovations of this study were ① Measure the peak systolic circumferential strain of endocardial, middle myocardial, and epicardial layers in patients with HCM. ② Measure the peak systolic radial strain in HCM patients. ③ Measure the peak systolic rotation degrees at the different short-axis levels in HCM patients, then to assess the changes in the left ventricular systolic function at the short-axis levels in HCM patients.

## Methods

### Ethical approvals

Recruitment to the study followed a full explanation of our methods including the fact that there was no risk of harm. Written informed consent was accepted. The Human Subjects Committee of Changzhou No. 2 People’s Hospital approved this study.

### Study sample

Thirty seven HCM patients and 41 age- and gender- matched healthy subjects were enrolled for the research. The diagnosis of HCM was based on the transthoracic echocardiography findings, and the inclusion criteria were as follows [20]: M-mode and/or 2D echocardiographic evidence of wall thickness ≥ 15 mm in one or more LV myocardial segments and non-dilated left ventricle (LV). All enrolled HCM patients were had septal wall hypertrophy and with/without other LV walls hypertrophy, in the absence of another cardiac disease causing LVM hypertrophy, such as hypertensive heart diseases, aortic valve stenosis. The normal subjects had no evidence of family histories of HCM, hypertension, and any other diseases. All of the physical examination tests, the electrocardiogram and the echocardiography were normal.

### Conventional 2D Doppler echocardiography

Thirty seven HCM patients and 41 normal subjects all had conventional 2D Doppler echocardiography (Vivid E9, GE Healthcare, Horten, Norway), Left atrial diameter (LAD), interventricular septal thickness in end-diastolic period (IVSD) and LV posterior wall thickness in end-diastolic period (LVPWD) were measured in the parasternal long axis view of the left ventricular by M-mode. Biplane Simpson’s method was used to measure the LV ejection fractions (LVEF). The peak velocities during early diastole (Ve) and late diastole (Va) of the anterior mitral valve were measured by pulsed-wave Doppler, and the ratio of Ve/Va was calculated. ECG leads were connected to each individual in all groups. Hold on the breath, standard high frame rate (60–90/s) of the parasternal short-axis views at the base, middle and apex of three consecutive were acquired for offline analysis.

### Data analysis for LV systolic function

We analysis the short-axis views at the base, middle and apex using 2D–STI software (2D–Strain, EchoPac PC version 113, GE Healthcare, Horten, Norway). Used the button SAX-MV, SAX-PM and SAX-AP to sketched the endocardial, respectively, then the software would create a region of interest (ROI) automatically which contained endocardial, middle myocardial and epicardial layers, then adjusted the ROI to make the myocardium included well. Approved the ROI, the software would divide the LV into six segments, and then the peak systolic circumferential strain of endocardial, middle myocardial and epicardial layers, the peak radial systolic strain and the different short-axis rotation degrees could be calculated and recorded.

### Statistical analysis

All of the analysis was performed using a commercially available package (SPSS 17.0. SPSS Inc., Chicago, IL, USA). Whether the distribution of the data in all subjects was normal were assessed by Kolmogorov-Smirnov’s test. If the data distribution was normal, differences between HCM patients and normal subjects were compared with an independent student t-test, for variables with a non-normal distribution, the nonparametric Mann-Whitney test was used. The correlation between the IVSD and the peak circumferential systolic strain of endocardial, middle myocardial, and epicardial layers, the radial systolic strain used the correlations test. Pearson correlation was used if the data distribution was normal, however, Spearman correlation was chosen if the data distribution was non-normal distribution. Data were presented as the mean ± s.d.. Difference was considered statistically significant in all tests when the *P*-value was less than 0.05.

## Results

### Basic information in HCM patients and the normal subjects

The values of LAD, IVSD and LVPWD in HCM patients were larger than normal subjects (*p* < 0.001). There were no significant difference in LVEDV, LVESV, LVEF, Ve, Va and Ve/Va (*p* > 0.05) (Table 1).

**Table 1:** The basic Information in HCM patients and control subjects from conventional Two-Dimensional Doppler Echocardiography (mean ± s.d)

	HR (bpm)	LAD (mm)	IVSD (mm)	LVPWD (mm)	LVEDV (ml)	LVESV (ml)	LVEF (%)	Ve (m/s)	Va (m/s)	Ve/Va
HCM (37)	72 ± 13	42 ± 5	19 ± 4	10 ± 1	80 ± 17	27 ± 9	67 ± 6	0.75 ± 0.16	0.62 ± 0.23	1.39 ± 0.57
Normal (41)	72 ± 12	35 ± 3	9 ± 1	9 ± 1	80 ± 12	29 ± 7	65 ± 6	0.84 ± 0.15	0.69 ± 0.18	1.29 ± 0.39
*P*-Value	0.900	**< 0.001**	**< 0.001**	**< 0.001**	0.845	0.354	0.137	0. 207	0.143	0.367

*LAD* left atrial diameter, *HR* heart rate, *IVSD* interventricular septal thickness in end-diastolic period, *LVPWD* left ventricular posterior wall thickness in end-diastolic period, *LVEDV* left ventricular end-diastolic volume, *LVESV* left ventricular end-systolic volume, *LVEF* left ventricular ejection fraction, *Ve* the peak velocity during early diastole of anterior mitral leftlet, *Va* the peak velocity during late diastole of anterior mitral leftlet

bold number is specify the significance of the comparision

### The peak systolic circumferential strain in different myocardium layers

**Fig. 1:**
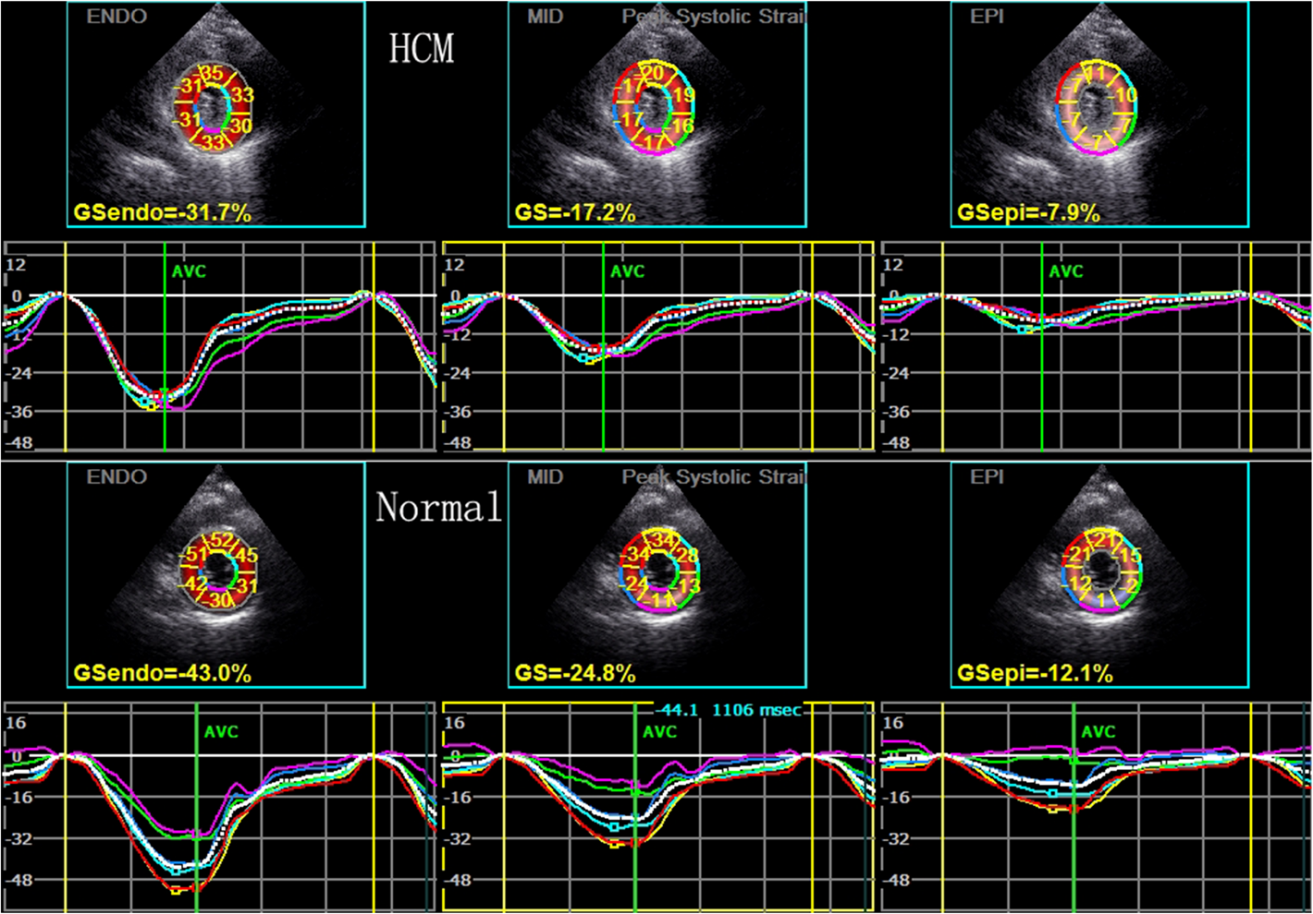
The peak systolic circumferential strain of the endocardial, the middle and the epicardial layers of left ventricular in HCM patients and normal subjects

The trend of the peak systolic circumferential strain of endocardial, middle myocardial, and epicardial layers of all the subjects was: endocardial > middle myocardial > epicardial. The strain absolute values of the anter-septum and anterior walls (in all layers) in HCM patients had significant lower than normal subjects. The strain absolute values of the septum wall (middle and epicardial layers) in HCM patients had significant lower than normal subjects. The strain absolute values of the posterior wall (endocardial and middle layers) in HCM patients had significant larger than normal subjects. Although the other walls had no significant difference, the absolute values of HCM patients were larger than normal subjects. (Table 2, Fig. 1).

**Table 2:** Comparision of the peak systolic circumferential strain of endocardial, the middle myocardial and epicardial layers and peak systolic radial strain in HCM patients and control subjects (mean ± s.d.)

LV Walls	PSCS (%)									PSRS (%)		
	Endocardial			Middle myocardial			Epicardial					
	HCM (37)	Normal (41)	*P*-value	HCM (37)	Normal (41)	*P*-value	HCM (37)	Normal (41)	*P*-value	HCM (37)	Normal (41)	*P*-value
Ant-Septum	**−29.16 ± 6.26**	**−34.16 ± 5.42**	**< 0.001**	**−16.79 ± 4.58**	**−23.62 ± 4.62**	**< 0.001**	**−9.36 ± 3.83**	**−15.95 ± 4.29**	**< 0.001**	**23.89 ± 9.82**	**40.22 ± 12.50**	**< 0.001**
Anterior	**−24.73 ± 6.43**	**− 28.23 ± 7.01**	**0.025**	**−13.92 ± 4.61**	**−18.83 ± 5.53**	**< 0.001**	**−7.24 ± 3.73**	**−11.91 ± 4.70**	**< 0.001**	**27.12 ± 9.75**	**41.06 ± 11.92**	**< 0.001**
Lateral	−22.33 ± 5.32	−20.95 ± 6.45	0.310	−12.45 ± 3.25	−12.58 ± 4.56	0.884	−6.18 ± 2.58	−6.61 ± 3.82	0.568	33.65 ± 11.52	**42.27 ± 11.73**	**0.002**
Posterior	**−22.62 ± 8.15**	**−17.66 ± 7.45**	**0.006**	**−13.20 ± 4.95**	**−10.58 ± 4.89**	**0.021**	−7.11 ± 3.22	−5.60 ± 3.60	0.055	**37.25 ± 13.33**	**43.26 ± 11.59**	**0.036**
Inferior	−26.47 ± 7.79	−23.87 ± 7.64	0.141	−15.89 ± 5.37	−14.93 ± 5.46	0.437	−8.94 ± 4.12	−8.50 ± 4.16	0.639	35.66 ± 14.20	**43.59 ± 11.83**	**0.009**
Septum	−31.73 ± 7.12	−34.08 ± 6.58	0.134	**−19.68 ± 5.00**	**−23.53 ± 5.28**	**0.002**	**−12.08 ± 4.14**	**−15.75 ± 4.89**	**< 0.001**	**27.88** ± **11.55**	**42.39 ± 12.63**	**< 0.001**

*PSCS* peak systolic circumferential strain, *PSRS* peak systolic radial strain

bold number is specify the significance of the comparision

### The peak systolic radial strain

**Fig. 2:**
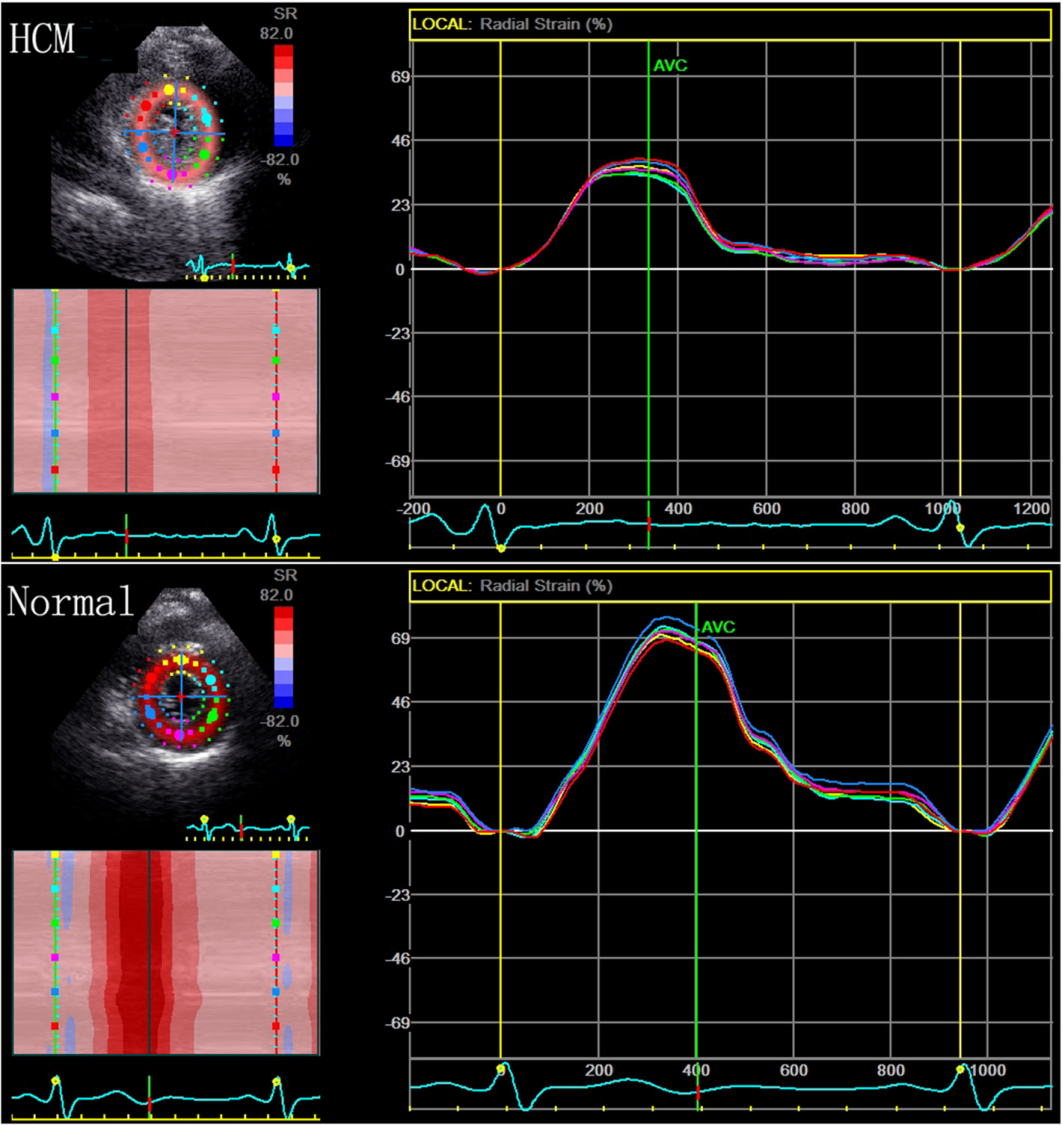
The peak systolic radial strain of left ventricular in HCM patients and normal subjects

All of the peak systolic radial strain in HCM patients was significantly lower than normal subjects. (Table 2, Fig. 2).

### Rotation degrees at different short-axis levels

In the systolic period, the LV apex wall rotated counter-clockwise, whereas the LV basal wall rotated clockwise in all subjects. Middle LV rotation was clockwise in HCM patients. The absolute values of peak systolic rotational degrees in the basal and middle short-axis levels in HCM patients had significant larger than normal subjects. (Table 3).

**Table 3:** The rotational degrees at different short-axis levels in HCM patients and normal subjects (mean ± s.d.)

	Rotational degrees in different short-axis levels		
	Basal (°)	Middle (°)	Apex (°)
HCM (37)	−11.27 ± 5.14	−3.90 ± 4.86	4.84 ± 7.34
Normal (41)	−4.65 ± 6.65	−0.87 ± 5.98	6.42 ± 7.35
*P*-Value	**< 0.001**	**0.017**	0.346

bold number is specify the significance of the comparision

### The correlation between IVSD and the peak systolic circumferential strain in different myocardium layers, the peak systolic radial strain

**Fig. 3:**
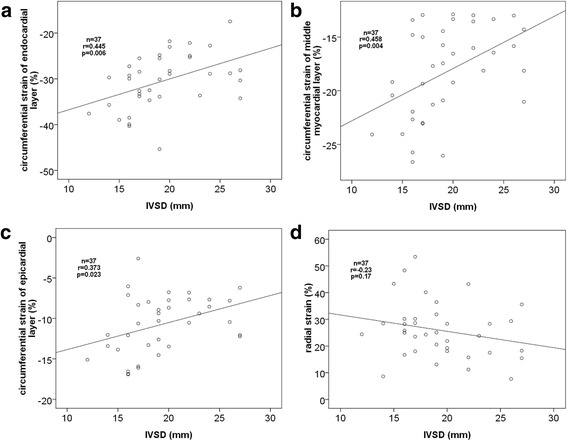
The correlation between IVSD and the peak systolic circumferential strain of the endocardial (**a**), the middle (**b**) and the epicardial layers (**c**), the peak systolic radial strain (**d**) in HCM patients. IVSD: Interventricular Septal Thickness in end-diastolic period

The IVSD correlated well to the peak systolic circumferential strain of endocardial, middle myocardial, and epicardial layers of the septum wall (Endocardial: *r* = 0.445, *p* = 0.006, Middle myocardial: *r* = 0.458, *p* = 0.004, Epicardial: *r* = 0.373, *p* = 0.023). There was no correlation between IVSD and the peak systolic radial strain (*r* = − 0.230, *p* = 0.170) (Table 4, Fig. 3).

**Table 4:** The correlation between IVSD and the peak systolic circumferential strain of endocardial, the middle myocardial and epicardial layers and peak radial strain in HCM patients

	PSCS			PSRS
	Endocardial	Middle	Epicardial	
*r*-value	0.445	0.458	0.373	−0.230
*p*-value	**0.006**	**0.004**	**0.023**	0.170

*PSCS* peak systolic circumferential strain, *PSRS* peak systolic radial strain

bold number is specify the significance of the comparision

## Discussion

HCM is a genetic disease, and mainly was characterized by left ventricular hypertrophy [21]. The systolic function detects by conventional echocardiography like LVEF is often normal, so the subclinical LV dysfunction cannot be identified by 2D conventional echocardiography. Peak short-axis systolic myocardial strain detect by 2D–STI can reflect the systolic function accurately. As we know, the peak circumferential strain of endocardial, middle myocardial, and epicardial layers in HCM patients is little reported.

Tigen K et al. [22] detected the LV systolic function by measuring the circumferential strain of the HCM patients, and found that the circumferential strain was significantly lower in patients with HCM compared with those of normal subjects. From the results, we found that, in the hypertrophied LVM, the LV peak systolic circumferential strain was decreased, and in non-hypertrophied myocardium, the strain value was increased. In HCM patients, LV hypertrophy, myocardial fibrosis, fiber disarray in the LV myocardium maybe a reason for the results. Once the LV was hypertrophy, the sequence of endocardial, middle myocardial, and epicardial layers had changed. The segmental systolic function was impaired. In order to maintain the normal LV systolic function, the non- hypertrophied myocardium enhanced their peak systolic circumferential strain. HCM patients with non- hypertrophied myocardium appeared to compensate for the early systolic changes via increased circumferential strain in order to keep the normal LV systolic function.

Yajima R et al. [8] found regional peak radial strain in basal, middle and apical levels were all significantly lower in HCM subjects than those in normal subjects. Our results were according to their results. Peak systolic radial strain in HCM patients was significantly lower than in the normal subjects. In HCM patients, decreased LV peak systolic radial strain appears not only in hypertrophied LVM, but also in non-hypertrophied myocardium. Peak systolic radial strain can reflect the systolic function very conveniently and accurately.

Zhang HJ et al. [23] investigated whether left ventricular twist analysis can detect the extent of myocardial fibrosis in patients with HCM, and found that, left ventricular twist mechanics are associated with the extent of myocardial fibrosis, and LV-twist assessment by STI may be clinically useful. Carasso S et al. [24] found middle LV rotation was clockwise (opposite to normal), they found that, in both HCM and normal subjects, LV rotation, viewed from the apex, was clockwise at the base, and count-clockwise at the apex, the difference was in the middle level. Ni XD et al. [25] found that in normal subject the transition from basal clockwise rotation to apical counterclockwise rotation is located at the papillary muscle level. Our research was according with the previous studies. The base-to-apex twist plane in HCM patients was changed. The peak rotational degrees at the base and middle short-axis levels in HCM patients were larger than normal subjects. Sengupta PP et al. [26] told us in the LV myocardial wall, the myofibers geometry changes smoothly from a right-handed helix in the endocardium to a left-handed helix in the epicardium such that the helix angle varies continuously from positive at the endocardium to negative at the epicardium. When the myofibres contract and relax, the cardiac have three motions: longitudinal, circumferential and radial, also produced the rotational motion. The pathogenesis of abnormal rotation at the middle level and the different rotation degrees are not clear. When the LV fibrosis, hypertrophied and stiffening, the LV myofibres were remodeling, the original balance of endocardial, middle myocardial, and epicardial myofibres was changed. Because the longitudinal and radial function were decreased in HCM patients, so for another possible reason was, in order to keep normal LV systolic function. HCM patients enhanced the peak rotational degrees at the base and middle short-axis levels.

The IVSD correlated well to the peak circumferential systolic strain of endocardial, middle myocardial, and epicardial layers of the septum wall, we concluded that, the more thickening of IVSD, the more circumferential systolic function was impaired. Through the correlation analysis, we found that the thickening of IVSD in HCM patients was consistent with its systolic function. There was not any correlation between IVSD and the peak systolic radial strain of the septum wall (*r* = − 0.230, *p* = 0.170), we concluded that, the thickening of IVSD had no internal relationship with peak systolic radial strain.

## Conclusions

According to this research, we know the short-axis systolic function is impaired despite the presence of preserved LVEF in patients with HCM. The peak circumferential systolic strain of the different layers, peak systolic radial strain and rotation degrees of the different short-axis levels detected by 2D–STI are very sensitive for assessing the systolic function in HCM patients. In the clinical implication, the study can help us to know the early cardiac dysfunction of HCM patients, then to give them early treatment and assess the effect after the treatment.

### Limitations

The greatest limitation of this study is that the relationship of decreasing short axis functions to a clinical outcome or event is not investigated. Another important limitation is the small number of patients. The third limitation is that we don’t evaluate the relationship with other image techniques, such as cardiac MR, SPECT.

## Abbreviations

2D–STI: 2-dimensional speckle tracking imaging
HCM: Hypertrophic cardiomyopathy
IVSD: Interventricular septal thickness in end-diastolic period
LAD: Left atrial diameter
LVEDV: Left ventricular end-diastolic volume
LVEF: Left ventricular ejection fraction
LVESV: Left ventricular end-systolic volume
LVH: Left ventricular hypertrophy
LVPWD: LV posterior wall thickness in end-diastolic period
Va: The peak velocities during late diastole of the anterior mitral valve
Ve: The peak velocities during early diastole of the anterior mitral valve
